# The role of [18F]FDG-PET/CT in gram-positive and gram-negative bacteraemia: A systematic review

**DOI:** 10.3389/fnume.2022.1066246

**Published:** 2022-11-18

**Authors:** Alice Packham, Niamh Spence, Tanveer Bawa, Rohit Srinivasan, Anna L. Goodman

**Affiliations:** ^1^ Department of Infectious Diseases, Guy's and St Thomas' NHS Foundation Trust, London, United Kingdom; ^2^ Department of Radiology, Guy's and St Thomas' NHS Foundation Trust, London, United Kingdom; ^3^ MRC Clinical Trials Unit, University College London, London, United Kingdom

**Keywords:** clinical diagnostics and imaging, bloodstream infection bacteraemia, sepsis - diagnostics, 18-FDG PET/CT, gram positive, gram negative, systematic reviews, mortality

## Abstract

**Objectives:**

Bacteraemia is associated with significant morbidity and mortality. [18F]FDG-PET/CT is increasingly used to detect infectious metastatic foci, however there remains international variation in its use. We performed a systematic review assessing the impact of [18F]FDG-PET/CT in adult inpatients with gram-positive and Gram-negative bacteraemia.

**Design:**

The systematic review was performed according to PRISMA guidelines. Studies published between 2009 and December 2021 were searched in MEDLINE, EMBASE and Cochrane clinical trials database. Data extraction and quality assessment was performed using ROBINS-I and GRADE.

**Setting:**

Eligible study designs included randomised-controlled trials, clinically-controlled trials, prospective trials, retrospective trials, case-control studies, and non-controlled studies.

**Participants:**

Studies solely assessing adult inpatients with blood-culture confirmed bacteraemia with one cohort of patients receiving [18F]FDG-PET/CT were included.

**Main outcome measures:**

primary outcomes were mortality, identification of metastatic foci and relapse rate. Studies not examining any of the pre-specified outcomes were excluded.

**Results:**

Ten studies were included, of which five had a non-PET/CT control arm. Overall, there was low quality of evidence that [18F]FDG-PET/CT is associated with reduced mortality, improved identification of metastatic foci and reduced relapse rate. Six studies assessed *Staphylococcus aureus* bacteraemia (SAB) only; nine studies included Gram-positive bacteraemia only, and one study included data from Gram-negative bacteraemia. Two studies compared outcomes between patients with different types of bacteraemia. Four studies identified a statistically significant difference in mortality in [18F]FDG-PET/CT recipients and controls. Relapse rate was significantly reduced in patients with SAB who received [18F]FDG-PET/CT. Studies identified significantly higher detection of metastatic foci in [18F]FDG-PET/CT recipients compared to controls. [18F]FDG-PET/CT was the first to identify an infectious site in 35.5% to 67.2% of overall foci identified.

**Conclusions:**

Further research is required to establish the role of [18F]FDG-PET/CT in bacteraemia, and its impact on management and mortality.

## Introduction

A diagnosis of bloodstream infection is a major cause of morbidity and mortality (1). Source identification is complicated by varied clinical presentation, and patients often present without localising symptoms (2). An infectious focus is not identified in up to 20% of bacteraemia cases, suggesting low sensitivity of current investigations (2, 3). Failure to identify an infectious focus hinders accurate treatment decision-making (3) and is associated with a significant increase in case-fatality-rate (4).

Gram-positive bacteria are responsible for up to 65% of all bacteraemia cases. *Staphylococcus aureus* is the most common cause of gram-positive bacteraemia (5) and is often associated with metastatic infections (6). The incidence of gram-negative bacteraemia has recently increased considerably, with *Escherichia coli*, *Pseudomonas aeruginosa* and *Klebsiella* spp., the commonest causes (7, 8). While *Staphylococcus aureus* bacteraemia (SAB) is most frequently discussed as a cause of metastatic infectious foci, gram-negative bacteria also cause metastatic foci and bacteraemia of unknown origin (9, 10).

Positron emission tomography/computed tomography (usually ^[18F]^fluorodeoxyglucose (FDG)/PET/CT, subsequently denoted as PET/CT) is increasingly utilised to detect abnormal glucose metabolism in infection (11). In contrast to conventional imaging techniques, PET/CT enables whole-body detection of hypermetabolic foci. There remains wide international variation in the use of PET/CT in bloodstream infection, secondary to diagnostic guidelines and scanner accessibility (12, 13). However, its use for the detection of infectious foci in bacteraemia is promising. PET/CT has recently been incorporated into the European guidelines for diagnosis for prosthetic valve endocarditis and CIED infections (14).

We performed a systematic review assessing the available evidence of the impact of PET/CT on mortality, identification of metastatic foci and clinical outcomes in adult inpatients with bacteraemia*.* We reviewed studies assessing the utility of PET/CT in both gram-positive and gram-negative bacteraemia, aiming to provide a broad, narrative perspective.

## Methods

### Study design and eligibility

This systematic review was performed in line with the Preferred Reporting Items for Systematic Reviews and Meta-Analyses (PRISMA) statement (15). The study was registered prospectively with PROSPERO (ID: CRD42021293352).

We included studies assessing outcomes following PET/CT in adults with blood culture-confirmed bacteraemia. Eligible study designs included primary evidence from randomised-controlled trials, clinically controlled trials, prospective trials, retrospective trials, case-control studies, and studies without a control arm. We excluded case studies and case series, studies with fewer than 30 patients and without full text available.

We aimed to assess multiple patient-related outcomes through a narrative review. Our main outcomes were mortality, time to discharge, microbiological recurrence of infection and clinical recurrence of infection. Additional outcomes included detection of metastatic infectious foci, duration of antibiotics, overall hospital stay length, re-admission, desirability of outcome ranking (DOOR) score for SAB, change in antibiotic course or delivery and mode of antibiotic delivery. Studies not examining any of the pre-specified outcomes were excluded.

### Search strategy, data extraction and analysis

A systematic search was performed of OVID Medline, OVID EMBASE and the Cochrane clinical trials database. The search included synonyms and MeSH headings for PET/CT, synonyms for bacteraemia, and source identification. Date was limited from 1st January 2009 to 1st December 2021. Full search details are in Supplementary Appendix S1. Manual search of www.clinicaltrials.gov was carried out for ongoing trials. A grey literature search was performed to identify any additional studies. Papers were screened in a two-stage process: title and abstract screening then full text screening. Two investigators independently evaluated all abstracts identified from the search based on pre-specified inclusion and exclusion criteria. Full paper review was carried out by two independent investigators. If no consensus was reached, a third investigator made a final decision. A standardised Excel spreadsheet was utilised for data extraction. Discrepancies were resolved through discussion.

### Quality assessment

Evaluation of risk of bias was carried out for each included study by two independent investigators. In the five studies with a no PET/CT comparator arm, the ROBINS-I tool was utilised (16). The ROBINS-I tool could not be fully applied to studies without a control arm, and they were deemed inherently at critical risk of bias due to their lack of comparator. For key outcomes, the Grading of Recommendations Assessment, Development and Evaluation (GRADE) framework was used to assess the certainty of the evidence.

## Results

### Search results

**Figure 1:**
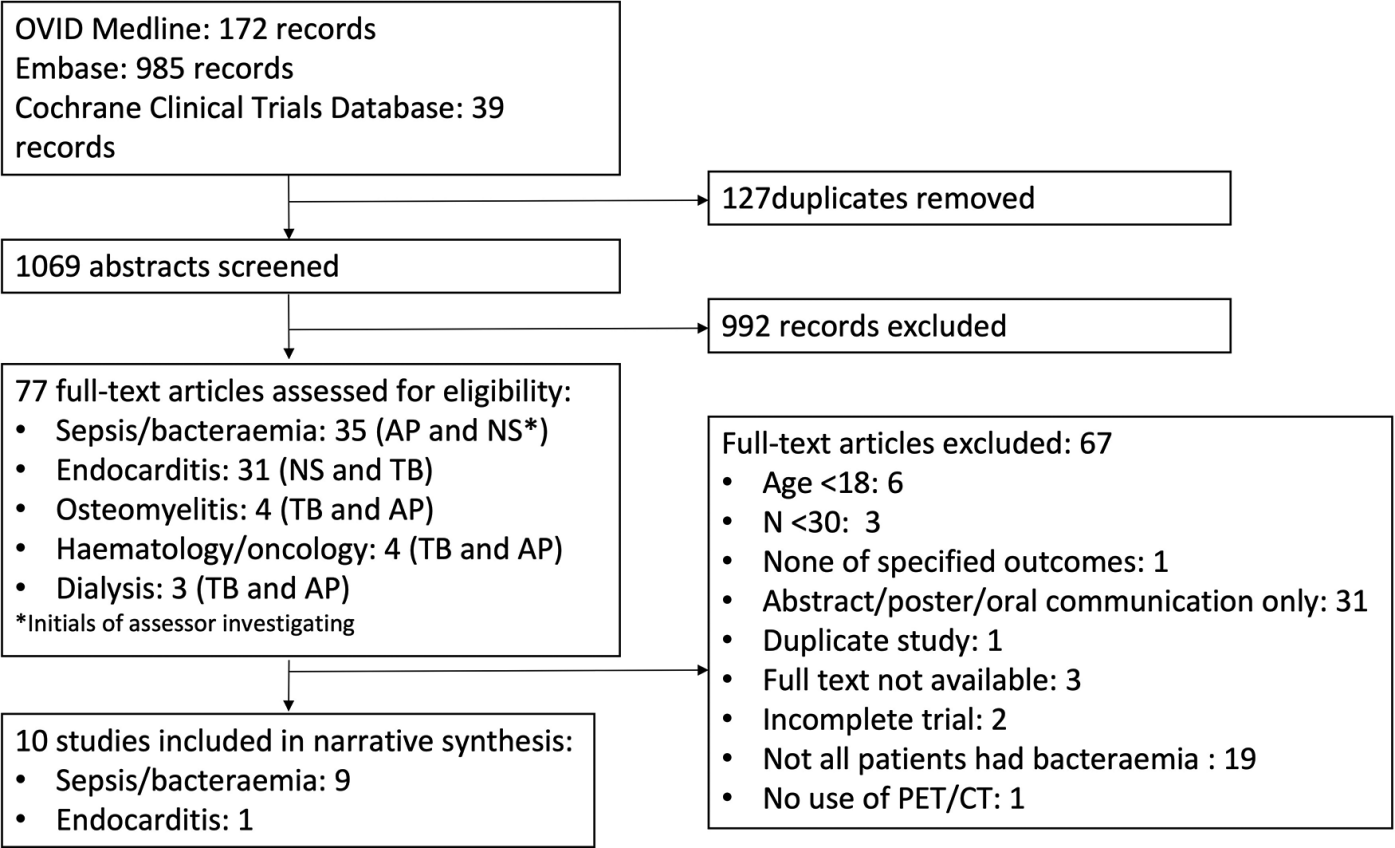
PRISMA flow diagram of study selection.

We identified 1196 records via database searches of Pubmed: 172 through OVID Medline, 985 through Embase and 39 through Cochrane Clinical Trials Database (Figure 1). We removed 127 duplicates, and screened 1,069 abstracts. We assessed 77 full-text articles for eligibility. 10 studies were eligible for the narrative synthesis. No published data meeting inclusion criteria was identified from manual search of www.clinicaltrials.org. (Figure 1).

### Characteristics of eligible, included studies

We identified 10 suitable studies (Table 1). They included 1,902 patients, of which 553 did not receive PET/CT. Eight studies were carried out in Europe (17–24) and the remaining two in Israel and Taiwan (25, 26). There were no randomised controlled trials. Five studies were non-randomised with a comparator arm of patients who did not undergo PET/CT (18, 19, 22, 24, 26). Of those without a non-PET/CT comparator arm, one study (17) retrospectively compared patient outcomes before and after the incorporation of an infectious disease structured bedside consultation during clinical work up, including associated numbers of PET/CT scans received in each cohort. Four further studies were observational studies without comparator arms (20, 21, 23, 25). Six trials included only retrospective patient data (17–20, 24, 25). Two used prospectively recruited patients only (21, 26). Two had prospective study arms with retrospective control arms (22, 23).

**Table 1:** Baseline features of 10 included studies.

First author, year	Country	Enrolment period	Study design	Population (adults only)	*n* patients^a^	%gram positive (G+)	of gram-positive, %SAB	%gram negative (G-)	Timing of PET/CT from blood culture (days, d)	Study summary and aims
Ariaans, 2018	Germany	2009–2017	Retrospective non-randomised cohort	SAB inpatients capable of structured bedside consultation (SBC)	613 SAB patients. 484/613 high-risk SAB. 227/484 (46.9%) received PET/CT	100%	100%	0%	NR	Incorporation of SBC in 2012. Compared results pre- and post-intervention, including impact of SBC on SAB survival, clinical work up and clinical findings.
Berrevoets, 2017	The Netherlands	2013–2016	Retrospective non-randomised cohort	SAB inpatients	184 (105 study, 79 control)	100%	100%	0%	Median 8d (mean 8.7d)	Compared high-risk SAB patients who received PET/CT and low-risk SAB as “no PET/CT” controls. Assessed diagnostic value of PET/CT for newly diagnosed metastatic infection, treatment modifications and outcome in patients with SAB.
Berrevoets, 2019	The Netherlands	2013–2017	Retrospective non-randomised cohort	SAB inpatients	76 (36 study, 40 control)	100%	100%	0%	Mean 8.4d (SD 3.8)	Compared high-risk SAB patients who received PET/CT which confirmed no infectious metastases, to low-risk SAB who did not undergo PET/CT. Assessed safety of shorter treatment duration in patients without metastatic disease.
Vos, 2010	The Netherlands	2005–2008 (study), 2000–2004 (control)	Prospective non-randomised (retrospective controls)	Inpatients with high-risk G + bacteraemia^b^	345 (115 study, 230 control)	100%	Study: 64% (73), Control: 64% (146)	0%	Median 7d (mean 6.8d)	Compared high-risk G + bacteraemia patients who received PET/CT and low-risk G + bacteraemia as ‘no PET/CT’ controls. Assessed relapse rate, 3 month mortality, 6 month mortality, modifications to treatment.
Gompelman, 2021	The Netherlands	2013–2020	Retrospective observational (no controls)	Inpatients with catheter-related SAB	43	100%	100%	0%	NR	Assessment of PET/CT findings of catheter-related thrombus, and extent to which PET/CT contributed to final diagnosis of septic thrombosis.
Tseng, 2013	Taiwan	2011–2012	Retrospective observational (no controls)	Inpatients with SUO, PET-CT <2 weeks of diagnosis	53	58% (31)	49.1% (26)	36% (19)^c^	<2 weeks from sepsis diagnosis	Aimed to assess value of PET/CT with SUO and define which of their patients most benefit from PET/CT use.
Vos, 2012	The Netherlands	2005–2008	Prospective observational (no controls)	Inpatients with high-risk G + bacteraemia^b^	115	100%^d^	73.9% (85)	0%	<2 weeks from first positive BC	Evaluated utility of PET/CT for detecting metastatic infectious foci in G + bacteraemia.
Yildiz, 2019	Belgium	2014–2017	Retrospective non-randomised cohort	High-risk SAB inpatients	102 (48 study, 54 control)	100%	100%	0%	<1 week^e^	Compared high-risk SAB patients who did or did not receive PET/CT (controls not matched). Aimed to identify factors associated with mortality rate.
Ghanem-Zoubi, 2021	Israel	2015–2019	Prospective matched cohort	SAB inpatients	299 (149 study^f^, 150 controls)	100%	100%	0%	8-13 (median 11) days after SAB diagnosis	Compared SAB patients who received PET/CT with matched ‘no PET/CT’ controls. Aimed to investigate the benefit of adding PET/CT into workup.
Kouijzer, 2013	The Netherlands	2005–2008	Prospective, interventional (no controls)	Inpatients with high-risk G + bacteraemia (excluded pneumococcus)	79 patients (7 later excluded due to low PET/CT quality)	100%	73.60%	0	<14 days of diagnosis	High-risk G + bacteraemia patients recruited and underwent ECHO and PET/CT. Aimed to assess sensitivity and specificity of PET/CT to diagnose endocarditis.

**Definitions:** PET/CT, [18F]FDG-PET/CT; SBC, structured bedside consultation; SUO, sepsis of unknown origin; SAB, Staphylococcus aureus bacteraemia; BC, blood culture; NVE, native valve endocarditis; NR, not reported.

**High-risk SAB definition:** signs of infection for >48 h before initiation of appropriate antibiotic treatment, community acquisition of the bacteraemia, fever >72 h or positive blood cultures >48 h after initiation of appropriate antibiotic treatment.

^a^ study is patients who underwent PET/CT, control is patients who did not undergo PET/CT.

^b^ SAB and *Streptococcus*, excluded *S. pneumoniae* or *Enterococcus*. Required at least one high-risk risk factor.

^c^ *Klebsiella* most common, at 10/19. 3 (6%) had multiple bacterial species - 1 *Citrobacter koseri*, *Enterococcus faecalis* and *E. coli*.

^d^ 11.3% haemolytic *Streptococcu*s, 14.8% *Streptococcus viridans.*

^e^ Does not specify timing from what event (e.g. whether hospital admission or confirmed bacteraemia).

^f^ 124 recruited in interventional study and 25 patients had PET/CT by clinical indication.

Nine studies included only gram-positive bacteraemias, of which six studies included only SAB (17–20, 24, 26). Four studies only included patients with risk factors for metastatic infections (21–24). Of gram-positive studies, all excluded *Streptococcus pneumoniae* bacteraemia patients (21–23). One study included gram-negative bacteraemia cases, 36% (n = 19) of their total cohort. Of these, 52.6% (10/19) was caused by *Klebsiella* (25).

The aims of the studies without PET/CT control arms were diverse. One included study aimed to evaluate the role of PET/CT in endocarditis diagnosis, comparing PET/CT diagnostic sensitivity and specificity to echocardiography, the gold-standard (21).

A non-uniform approach to measure timing of PET/CT was identified between studies' methods. Most measured time from first positive blood culture. Two did not specify the time-point of PET/CT in methods or results (17, 20). One stated PET/CT occurred within one week, however we are unclear if this was measured from hospital admission or positive blood culture (24). Of those who specified timing of PET/CT from diagnosis, all were within 14 days from diagnosis. Seven studies reported if their cohort included both hospital- and community-acquired bacteraemia (17–19, 22, 23, 25, 26).

### Outcomes

#### Mortality

All five studies with a “no PET/CT” comparator arm assessed mortality rate as primary outcome (18, 19, 22, 24, 26). Four of these studies identified a statistically significant difference in mortality rate at follow up (measured at either 28 days, three months, six months or one year) (18, 22, 24, 26). Only one of these four reported a time point where mortality was not statistically significant, at three months (*p* = 0.18). This study showed a statistically significant difference in mortality from six months (*p* = 0.014) (22). Multivariate analysis in one paper identified three factors significantly affecting mortality: PET/CT reduced the risk, while kidney failure and bacteraemia of unknown origin increased the risk (24). Berrevoets (2019) compared high-risk SAB inpatients without evidence of metastatic foci on PET/CT to low-risk controls who did not receive PET/CT. There was no significant difference in mortality rate between groups (*p* = 0.64) (19) (Table 2).

**Table 2:** Mortality, relapse rate and foci identification.

Name, year	Control arm without PET/CT	No. of patients who received PET/CT	Mortality	Relapse rate	Of patients who received PET/CT, proportion with metastatic foci	Proportion of patients with >1 metastatic foci	Further details of positive PET/CT scans	Treatment modifications due to PET/CT findings
Ariaans, 2018	No^a^	227 (47% risk of complicated SAB)	NR	NR	114/227 (50.2%)	NR	74/114 (64.9%) patients with positive PET scans revealed ‘not clinically suspected’ metastatic infection	NR
Berrevoets, 2017	Yes	105 (99 high-risk SAB)	**At 3 months:** PET/CT 12.1%, no PET/CT 32.7% (*p* = 0.003).	**At 3 months:** 0% with PET/CT, 3% without PET/CT^b^ (all high-risk SAB sub-group)	73/99 (73.7%) - high-risk SAB only	47/73 (64.4%)	52/73 (71.2%) no signs or symptoms before PET/CT	104 modifications in 74/105 patients
			In total: 18.5% (34/184)					
Berrevoets, 2019	Yes	36	**At 3 months:** PET/CT 19.4% no PET/CT 15.0% (*p* = 0.64)	**At 3 months**: case group 2.8%, control group 5%	0% (study inclusion requirement)	Not applicable	Not applicable	Not applicable
Vos, 2010	Yes	115	**At 3 months:** PET/CT 21.9%, no PET/CT 28.8% (*p* = 0.18)^b^.	**At 3 months:** With PET/CT 2.6%, without PET/CT 7.4% (*p* = 0.09).	78/115 (67.8%) vs. 35.7% of controls, *p* < 0.05	30/78 (38.5%) study, 22/82 (28.2%) controls	50% of patients with metastatic foci on PET/CT did not have suggestive symptoms. 44/124 (35.5%) foci identified on PET/CT were new (in 35/115 patients).	Study group underwent more ECHO (83% vs. 29%).^d^ 73/78 received antibiotics for >14 days. 6/22 endocarditis patients had treatment extended for metastatic focus.
			**At 6 months:** PET/CT 19.1%, no PET/CT 32.3% (*p* = 0.014).	**In SAB group:** with PET/CT 1.4%, without PET/CT 8.9% (*p* = 0.014)				
Gompelman, 2021	No	43	NR	NR	11/43 (26.6%) were possible septic thrombosis.	NR	NR	PET/CT ruled out septic thrombosis in 21/43 (48.8%), of which therapy shortened or converted IV to PO in 20/21 (95.2%).
Tseng, 2013	No	53	NR	NR	35/53 (66%) positive FDG-PET results	NR	NR	13/53 (25%): 4 focus drainage, 9 surgery.
Vos, 2012	No	115	**At 6 months:** 22.6% (26/115). 21/26 had present metastatic foci.	NR	Not clear (84/115 patients have metastatic foci, not stated what imaging modality identified foci)	30/115 (26.1%)	59% of patients with positive PET/CT had no signs/symptoms. 35/115 (30%) of infections were first identified on PET/CT.	Treatment duration: with metastatic foci 44d; without foci 15d
Yildiz 2019	Yes	48	PET/CT vs. no PET/CT: **At 1 month:** *p* = 0.001, **at 3 months:** *p* = 0.004.	NR	22/48 (45.8%)	13/22 (49 foci total identified on PET/CT). 13 foci in control group (*p* < 0.00001)	NR	NR
			Overall mortality at 1 year: 31.4% (32/102), of which PET/CT 16.6% (8/48) vs. no PET/CT 44.4% (24/54), *p* = 0.002.^c^					
Ghanem-Zoubi, 2021	Yes	149	PET/CT vs. no PET/CT: At 1 month: 4% vs.13% (*p* = 0.004).	**At 6 months:** 0.7% higher in PET/CT group (not significant)^e^	**107 episodes (70.8%)**	41/107 (38.4%)	72/107 (67.2%) of infections had not been identified prior to PET/CT	Treatment duration significantly longer in PET/CT group (42d vs. 19d). Focus control interventions following PET/CT occurred in 27/151 episodes (17.9%)
			**At 3 months:** 13.9% vs. 28.5% (*p* = 0.004, in multivariate analysis odds ratio [OR], 0.39; 95% confidence interval [CI], 0.18–0.84; *p* = 0.016).					
			**At 6 months:** 13.9% vs. 28.5% (*p* = 0.002).					
Kouijzer, 2013	No	72	**6-month mortality:** with IE 22%, without IE 20% (*p* = 1.00). 50% mortality without IE but with increased heart valve 18F-FDG uptake vs. 18% without IE with normal 18F-FDG uptake (*p* = 0.18).	3/79 (3.8%) - 1 in definite IE, 2 in rejected IE	51/72 (70.8%)	NR	51/79 (64.6%) foci did not have associated suggestive symptoms. Endocarditis diagnosis by PET/CT: sensitivity 39%, specificity 93%, PPV 64%, NPV 82%	NR

**Abbreviations:** PET/CT, [18F]FDG-PET/CT; IE, infective endocarditis; IV, intravenous; PO, oral; NR, not reported.

^a^ Not all received PET/CT, no specific ‘no PET’ control arm.

^b^ Data obtained from Buis (2021) review (who asked the study authors directly).

^c^ Kaplan-Meier method.

^d^ If you remove endocarditis cohort, difference in those with metastatic foci is still significant (*p* < 0.0001).

^e^ relapse defined as a clinical relapse of infection within 6 months after treatment end with phenotypically identical S. aureus isolate from any site (excluding colonization).

Overall mortality was measured in two observational studies. Vos (2012) identified a six-month mortality rate of 22.6% (26/115) in bacteraemia patients who had received PET/CT. This paper identified significantly higher mortality rates in those with persistent positive blood cultures for over 48 hours (h) (*p* = 0.05), nosocomial acquisition (*p* = 0.03), and age >60 years (*p* < 0.01) (23).

Koujzer (2013) assessed patients who had received PET/CT and echocardiogram and either did or did not have suspected infective endocarditis (IE) according to the revised Duke's criteria. They outlined 50% mortality in patients without IE (as per revised Duke's criteria) but with increased heart valve 18F-FDG uptake, compared to 18% mortality in patients without IE and with normal 18F-FDG uptake. This was not statistically significant (*p* = 0.18) (21).

#### Relapse rate

Relapse rate was commented on by four studies (18, 19, 21, 26). Vos (2010) identified a significant difference in three-month relapse rate when analysing three-month mortality in SAB patients alone (PET/CT 1.4% vs. no PET/CT 8.9%, *p* = 0.04) (22). Two other studies commented on non-statistically significant reduced relapse rate in PET/CT group in SAB patients (0% vs. 3; and 2.8% vs. 5%, *p* = 1.00) (18, 19). Koujzer (2013) commented on an overall relapse rate of 3/79 (3.8%). We are not clear on the specific follow-up period of relapse (21) (Table 2).

#### Identification and location of foci by PET/CT

Nine studies discussed detection of metastatic infectious foci on PET/CT (17, 18, 20–26). The proportion with metastatic infectious foci ranged from 45.8% to 73.7%. The proportion of positive scans with multiple metastatic foci ranged from 35.7% (23) to 64.4% (18). Two studies compared detection of metastatic foci in PET/CT recipients compared to controls. One investigated high-risk SAB and the other included both gram-positive and gram-negative bacteraemias (22, 24). Both found significantly higher detection of metastatic foci in PET/CT recipients compared to controls (*p* < 0.05 and *p* < 0.00001) (Tables 2, 3).

**Table 3:** Location of infectious foci.

	Ariaans, 2018		Berrevoets, 2017		Vos, 2010^a^		Gompelman, 2021		Tseng, 2013		Yildiz, 2019		Ghanem-Zoubi, 2021		
	Total	first found on PET/CT (% total foci)	Total	first found on PET/CT (% total foci)	Total	first found on PET/CT (% total foci)	Total	first found on PET/CT (% total foci)	Total	first found on PET/CT (% total foci)	Total	first found on PET/CT (% total foci)	Total	first found on PET/CT (% total foci)	
No. (%) of patients with foci identified on PET/CT	114/227 (50.2%)		73/99 (73.7%) high-risk SAB only		78/115 (67.8%) vs. 35.7% of controls, *p* < 0.05		23/43 (54%)		35/53 (66%)		22/48 (45.8%)	Not reported	107/151 (70.8%)	72/107	**Totals**
Bone/joint (of which prosthesis)	46 (7)	20 (43.5%)	19 (NR)	7 (36.8%)	19 (9)	6 (31.6%)	NR (part of osteomyelitis)		2 (NR)		16 (NR)		58	31	160
Osteomyelitis (of which vertebral or spondylodiscitis)	46 (35)	29 (63%)	24 (14)	12 (50%)	17 (11)	9 (52.9%)	4 (NR) - recorded as osteo-myelitis/-arthritis		13 (13)		12 (12)		NR - recorded as bone and joint		116
Lung	NR		31	17 (54.8%)	12	6 (50%)	15		12		5		32	28	107
Skin and soft tissue	NR		31	20 (64.5%)	11	4 (36.4%)	3		0				35	15	82
Endovascular	5	3 (60%)	20	15 (75%)	20	12 (60%)	3		0		3		20	17	71
Cardiac (of which endocarditis)	10 (7)^a^	4 (40%)	18 (18)	11 (61%)	21 (21)	0 (0%)	NR		1 (1)		1 (1)		9 (NR)	2	60
Abdominal - includes liver, gallbladder, intra-abdominal (of which spleen)	NR		8 (5)	7 (87.5%)	6 (1)	2 (33.3%)	NR		2		1 (0)		9 (NR)	9	26
Muscular (of which psoas abscess)	NR (NR)		NR (7)		NR (3)		NR (0)		4 (2)		9 (5)		16 (0)	16	39
CNS (of which brain)	NR		NR		11 (NR)	3 (27.3%)	NR		0		0		0	0	11
Pericarditis, mediastinitis	NR		4	3 (75%)			NR		0		1		0	0	5
Kidney	NR		1	1 (100%)	1	0 (0%)	NR		1		1		NR	0	4
Orbital	NR		NR		3	0 (0%)	NR		0		0		0	0	3
Other/not specified	50	39 (78%)	NR		0	N/A	2 (excluding septic thrombosis)	0		0				52	
Total infectious foci	157		163		124		29		35		49		179		736

**Abbreviations:** PET/CT, [18F]FDG-PET/CT; MSK, musculoskeletal; NR, not reported.

^a^ Vos (2012) foci data not included because same patient foci data as Vos (2010). Data assessed from patients who received PET/CT including studies that documented foci sites only.

Two studies discussed PET/CT foci detection in patients without preceding clinical suspicion. These identified that the foci of 59% (23) and 73.7% (18) of patients with a positive PET/CT had not been clinically suspected. Four studies commented on the proportion of infections in which PET/CT was the first to identify an infectious site, following normal results from other imaging modalities (17, 22, 23, 26). This ranged from 35.5% (22) to 67.2% (26). Gompelman (2021) only included patients with catheter-related thrombus; in 85% of those diagnosed with a septic thrombosis, PET/CT was the deciding diagnostic factor (20).

Eight studies representing seven patient groups discussed the sites of metastatic infection. They reported 736 PET/CT scans (17–20, 22, 24–26). Importantly, some patient cohorts overlap due to multiple studies performed in a single patient group. Of these 736 scans, 61% of scans detected metastatic foci and a total of 736 infectious foci were identified. The three most identified site of metastatic foci were: osteomyelitis/bone and joint, lung, and skin and soft tissue. 7% of all metastatic foci reported were categorised as “other”.

Endocarditis diagnosis by PET/CT is complicated due to high cardiac uptake of 18F-FDG. Between the eight studies, 60 instances of cardiac foci were identified as metastatic foci on PET/CT. The included study specifically investigating the role of PET/CT in the diagnosis of endocarditis identified that a diagnosis of IE (by expert consensus) was made in 64% with increased PET uptake at heart valves, and 18% of those without increased uptake (*p* < 0.01) (21). Vos (2010) reported significantly more endocarditis identified in study patients than controls (*p* = 0.01). Of those with endocarditis, over 50% had a second metastatic focus detected in both PET/CT and non-PET/CT groups (22).

Vos (2012) included patients with SAB and risk factors for metastatic infection. They identified several factors that significantly increased likelihood of metastatic foci detection: higher mean CRP levels on admission (*p* < 0.01); treatment delay >48 h (*p* < 0.01) and unknown portal of entry (OR 5.6) (23).

#### Bacteraemia types

Four studies included patients with non-SAB (21–23, 25), three of which only investigated gram-positive bacteraemia (21–23). One included gram-negative bacteraemia (25). Two studies compared PET/CT findings across different bacteraemias. Vos (2012) identified similar rates of metastatic PET/CT findings between *Streptococcus* and SAB infection. However, they identified that pulmonary foci were more common in SAB than streptococcal bacteraemia (*p* = 0.01) (23). They also identified an unknown portal of entry as a significant risk factor for metastatic foci, and unknown portal of entry was significantly more likely in S*treptococcus* than SAB infection (*p* = 0.04). Tseng (2013) similarly identified no significant difference in PET/CT findings between gram-positive, gram-negative or polymicrobial infections (*p* = 0.741) (25).

### Risk of bias assessment and GRADE assessment

**Figure 2:**
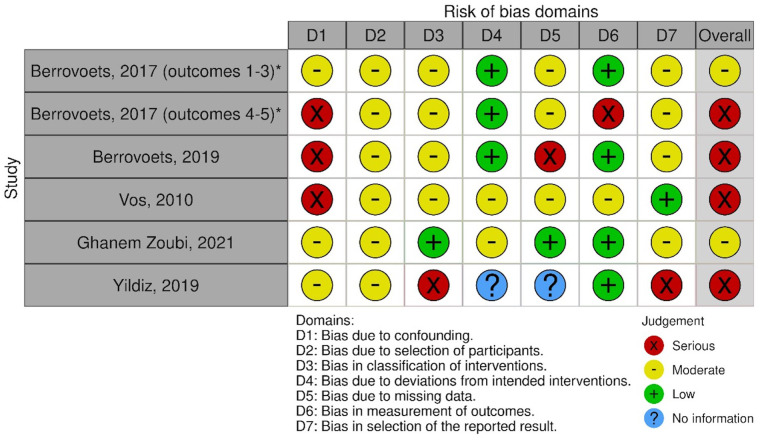
ROBINS-I risk of bias and GRADE assessment.

Of the five studies which included a comparator arm, we found one study at moderate risk of bias. One further study was judged at moderate risk for three of the study protocol's outcomes, and serious for two other outcomes. We felt that three studies were at serious risk of bias. A visual summary is shown in Figure 2 (27). A detailed outline of our bias assessment method and results is available in Supplementary Appendices S2, 3. Studies without a comparator arm were considered at critical risk of bias.

GRADE assessment was performed for four outcomes: mortality, relapse rate, ability of PET/CT to identify metastatic foci and treatment modifications due to PET/CT findings. For all four outcomes, the evidence was judged “very low”. This resulted from factors including: lack of randomised controlled trials, several studies at serious or critical risk of bias, heterogeneity in methodology between different studies, and focus on gram-positive bacteraemia (with limited evidence regarding gram-negative or anaerobic organisms).

## Discussion

Overall, our results show that there is a low certainty of evidence that PET/CT is associated with reduced mortality, identification of metastatic foci, and reduced relapse rate. Notably, none of the included papers discussed several key outcomes we aimed to assess. Our review was therefore limited to the outcomes reported in the literature. The number of studies investigating each outcome is low and they were all at some risk of bias.

Despite the low-quality evidence, studies tended to identify a benefit resulting from performance of PET/CT. Mortality was significantly lower in those who received PET/CT compared to those who did not (18, 22, 24, 26). Importantly, as PET/CT is solely a diagnostic tool, benefit on clinical outcome is determined by changes to clinical management resulting from PET/CT findings. One interesting study investigated outcomes of SAB patients who had no metastatic foci on PET/CT compared to those who did not receive PET/CT, identifying no significant difference in mortality (19). This is an important cohort requiring more investigation, particularly to help us identify in which patients it is safe to stop antibiotic treatment earlier.

A recent systematic review by Buis et al., 2021, analysed the impact of PET/CT on mortality in SAB (28). Their review included five studies in their qualitative synthesis, and required studies to have a control without PET/CT. It concluded that there was low certainty of evidence that PET/CT reduces mortality in patients with SAB. Mortality, while important (and our best evidenced outcome), is only one metric by which this can be measured. Appropriate PET/CT use may benefit wider outcomes including relapse rates, antibiotic decisions, and admission duration. These could all benefit allocation of limited healthcare resources. While these outcomes were mentioned in some included studies, overall, we identified heterogeneity in outcomes and method of assessment of PET/CT impact. The quality of evidence investigating these outcomes is very low. Future studies would benefit from incorporating control groups, with a consistent approach to assessing the impact of PET/CT.

To better focus limited PET/CT access, further studies should consider which patients are most likely to benefit from PET/CT application and should review a wider range of outcomes. Four papers identified in our review only included those with high-risk bacteraemia (21–24). While some studies compared high-risk SAB, general SAB and wider gram-positive bacteraemia, many excluded pneumococcus and only one study assessed gram-negative bacteraemia. Few separated their findings based on bacteraemia type, and only limited conclusions on outcomes based on causative bacterium can be drawn due to observational data and lack of matched cohorts. Overall, we identified little evidence regarding which organisms are most likely to seed, and to where. Several risk factors for a positive PET/CT finding were discussed, including unknown entry site, treatment delays, presence of foreign bodies and higher mean CRP (23). Additional investigation into these associations would help identify in which patient cohorts, and on which bacteraemia types, PET/CT may have the greatest impact.

The whole-body scanning of PET/CT enables detection of both infectious source and metastases, which is particularly important when foci are clinically silent. Nine studies discussed the detection of metastatic foci through PET/CT. There was heterogeny in how studies reported and categorised metastatic foci. This posed a challenge when comparing foci sites across studies. Common sites of metastasis included bone/joint, lung and soft tissue. Despite low quality evidence, between 35% (23) and 71% (18) of patients identified with metastatic foci did not have localising signs or symptoms. One paper commented on significantly improved detection of foci on PET/CT compared to other imaging modalities (24).

As previously discussed, the diagnostic capacity of PET/CT in endocarditis is complicated by high FDG uptake in cardiac muscle. One study specifically assessed the role of PET/CT in endocarditis diagnosis (21). Five further papers detected endocarditis as a metastatic focus on PET/CT. Vos (2010) identified that over 50% of patients with endocarditis in both PET/CT and no PET/CT groups had a second metastatic focus (22). Future studies assessing the role of PET/CT in detection of secondary metastatic foci in patients with proven endocarditis would further our understanding. Considering the potential for culture-negative endocarditis, and our requirement for confirmed bacteraemia in all patients, it is likely that our review excluded endocarditis papers which would aid discussion on this topic.

Our study had several limitations. Six of the 10 studies were carried out in The Netherlands, in the same tertiary centres (18–23). Multiple studies used retrospective cohorts which appear to use the same patient data sets (21–23). Studies had heterogenous outcomes which precluded a meta-analysis. Several studies included did not have a control group who did not receive PET/CT, making them inherently at critical risk of bias. Several relevant studies which may have provided further insight were excluded because a small proportion of patients were under 18.

Overall, large randomised controlled trials collecting a wider range of outcomes are needed to identify the precise role of PET/CT in SAB and bloodstream infections. A randomised-controlled trial investigating PET/CT in bacteraemia was registered on clinicaltrials.gov in 2018 and is assessing its role in SAB (TEPSTAR) (29). Further studies should be conducted across varied geographic locations to ensure findings can be applied to multiple healthcare systems. Studies would benefit from consistent approach to outcome measures such as mortality, determination of foci, or improved antibiotic stewardship to ensure the position of PET/CT in bacteraemia is fully examined.

## Data Availability

The raw data supporting the conclusions of this article will be made available by the authors, without undue reservation.
