# Supplementary material for: The role of [18F]FDG-PET/CT in gram-positive and gram-negative bacteraemia: A systematic review
Source: Front Nucl Med. 2022 Nov 18;2:1066246. doi: 10.3389/fnume.2022.1066246 (PMC11440864; doi:10.3389/fnume.2022.1066246)
Supplement: Supplementary file 1 [file Table1.docx]

**Appendix 1: search strategy**

**Ovid MEDLINE(R) ALL <1946 to November 23, 2021> and Ovid Embase <1974 to November 23, 2021>**

1. exp Positron-Emission Tomography/
2. PET.mp.
3. PET-CT.mp.
4. FDG-PET.mp.
5. FDG-PET-CT.mp.
6. 18F-FDG-PET.mp.
7. 18F-FDG-PET-CT.mp.
8. Fluorodeoxyglucose Positron Emission Tomography.mp.
9. 18F Fluorodeoxyglucose Positron Emission Tomography.mp.
10. 1 or 2 or 3 or 4 or 5 or 6 or 7 or 8 or 9
11. exp Bacteremia/
12. bacter?mia.mp.
13. septic?mia.mp.
14. sepsis.mp.
15. septic.mp.
16. bloodstream.mp.
17. blood stream.mp.
18. bloodstream infection.mp.
19. blood stream infection.mp.
20. 11 or 12 or 13 or 14 or 15 or 16 or 17 or 18 or 19
21. Detect*.mp.
22. Identif*.mp.
23. Workup.mp.
24. work up.mp.
25. diagnos*.mp.
26. 21 or 22 or 23 or 24 or 25
27. origi*.mp.
28. foc*.mp.
29. source.mp.
30. sources.mp.
31. metasta*.mp.
32. thromb*.mp.
33. embol*.mp.
34. 27 or 28 or 29 or 30 or 31 or 32 or 33
35. 10 and 20 and 26 and 34
36. limit 35 to yr="2009 -Current"


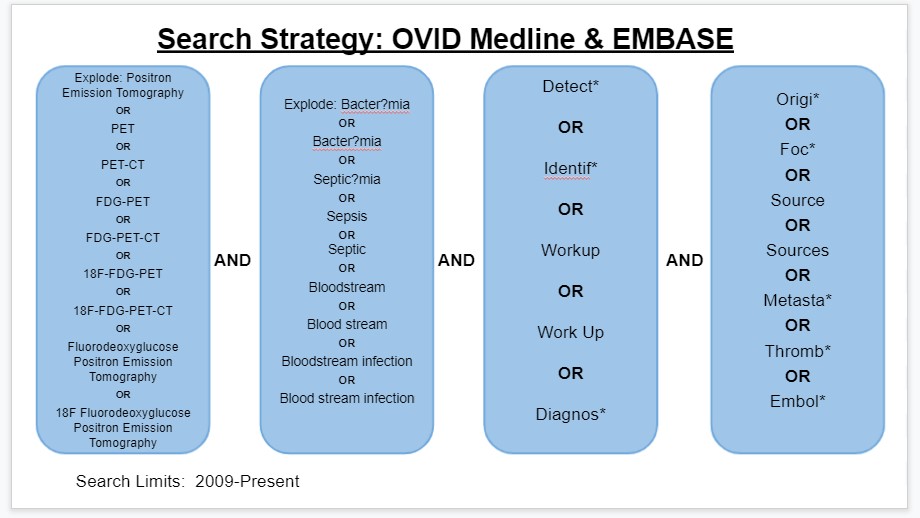


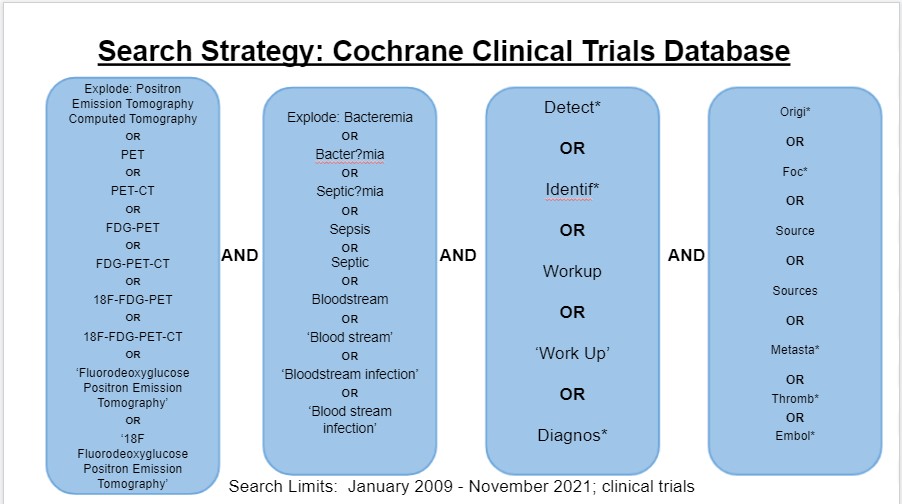


**Appendix 2: Risk of bias assessment methods**

*Specify a target randomized trial specific to the study:*

- **Design:** individually randomised
- **Participants:** hospitalised patients aged 18 or over with confirmed bacteraemia, defined as at least one positive blood culture
- **Intervention:** Use of ^18^ F-FDG PET/CT (PET/CT) imaging.
- **Comparator:** no use of PET/CT

*Aim of the study:*

- To assess the effect of assignment to the intervention, with the intervention being of proposed benefit

*Outcome of the study being assessed for risk of bias:*

**For each domain:**

1. **Bias due to confounding:**

- **Participant features:**
  - To assess whether participant demographics or comorbidities impact the likelihood of PET/CT being performed and therefore bias the estimated effect of PET/CT. Studies were required to outline their method for selecting participants for each arm.
- **Co-interventions:**
  - Bacteraemia management requires complex diagnostic and therapeutic interventions, particularly when considering of no known origin.
  - Diagnostic approach frequently requires various imaging modalities (e.g. echocardiography, magnetic resonance imaging) to identify potential sources of infection. Antibiotic treatment duration can be modified depending on clinical symptoms or results of these imaging modalities.
  - Management is often regulated by each site, therefore changes in hospitals or wards could increase risk of modified treatment plans.
  - Similarly, sites often update their clinical approach to bacteraemia. Therefore, studies using historical controls were also considered at risk of bias.
- **Time variant confounding:**
  - There is risk of bias if study or control participants are monitored for different periods of time depending on timing of intervention. Studies ought to ensure that participants who received PET had same total duration of follow up as those in control group e.g. time before PET/CT added to time after PET/CT must be equivalent to overall time of follow up in control group.
- **Grading of bacteraemia:**
  - Due to the range of presentations in bacteraemia, different sites use specific criteria to mark as ‘high risk’ or ‘low risk’. As above, going between different sites was classed as a risk of bias.
  - If PET/CT was allocated to those at ‘high risk’, this could either:
    - Give data that suggests that PET/CT increases risk of mortality, considering more severe infection
    - Miss severe cases, too ill to receive PET/CT, making PET/CT intervention arm appear to have lower risk of mortality.

1. **Bias in selection of participants into the study:**

- If participants experience outcomes (e.g. death from bacteraemia, compromising event that would prevent them from receiving PET) to be part of the study, this could again lead to immortal time bias. Studies needed to outline that this was recognised, and how it was adjusted or avoided.
- Studies needed to describe their inclusion and exclusion criteria to incorporate patients into the study

1. **Bias in classification of interventions:**

- For each participant, studies were required to outline the duration between first positive blood culture and PET/CT imaging, with reason for duration outlined. Where time length ranged significantly or was of longer duration (e.g. >2 months) this was considered at risk of bias.
- Studies should consider risk of immortal time bias, monitoring that everyone who is recruited is included, whether or not they receive the intervention (n/a if retrospective)

1. **Bias due to deviations from intended interventions:**

- Studies were required to report on patient preparation, execution of PET/CT and reporting of PET/CT. This included dietary planning: whether starved, considering recognised difficulty in assessing PET/CT following high carbohydrate meals.
- Papers which did not outline a consistent approach were deemed at risk of bias.

1. **Bias due to missing data:**

- Studies were required to outline duration of follow up, and include data from all patients who underwent intervention in primary analysis.
- For any missing data, for example reason for early termination of follow up in specific patients (for example lost to follow up or transfer to another site) this needed to be clearly stated.

1. **Bias in measurement of outcome:**

- Studies were required to outline clearly their primary and secondary outcomes.

1. **Bias in selection of the reported result:**

- Studies were required to provide results that was outlined in their original protocol as initially outlined.

**Appendix 3:** ROBINS-I results

**Article**: Berrevoets, 2017

Outcomes assessed:

1. Mortality at three months
2. Relapse of SAB at three months
3. Patient outcome at three months
4. Treatment modifications*
5. Newly diagnosed metastatic infection*

*The results for the bias assessment for these outcomes were different in certain domains ***(bias due to confounding*** *and* ***bias in the measurement of the outcome***), hence splitting the ‘assessment’ column for each domain into two. In all other domains the result was the same.

Assessors: AP, NS

| Risk of Bias Domain | Assessment | Comments |
| --- | --- | --- |
| Bias due to confounding. | Outcomes 1, 2, and 3: **Moderate** | - Clear outline of (and only includes individuals who had) high-risk SAB. - Does not specify how 99 individuals were allocated to [18F]FDG-PET/CT (only that it was a clinical decision at the time of treatment). - Statistical analysis defines use of multivariate analysis for potential confounders, and the Charlson comorbidity index to adjust for demographic composite variable. - Single-centre study over one time-period. |
|  | Outcomes 4 and 5: **Serious** | - Did not control confounding domains on assessment of these outcomes. |
| Bias in selection of participants. | Outcomes 1, 2, and 3: **Moderate** | - Inclusion criteria were clearly outlined. - Retrospective study, therefore measured all individuals who received the intervention. - A sensitivity analysis which excluded those who died <7 days from admission only measured three-month mortality. - Start of follow up and start of intervention did not coincide. |
|  | Outcomes 4 and 5: **Moderate** |  |
| Bias in classification of intervention. | Outcomes 1, 2, and 3: **Moderate** | - Median duration to [18F]FDG-PET/CT of eight days (mean 8.7 days). - Does not specify the duration to [18F]FDG-PET/CT for each individual patient, or the shortest / longest duration to [18F]FDG-PET/CT . |
|  | Outcomes 4 and 5: **Moderate** |  |
| Bias due to deviations from intended interventions. | Outcomes 1, 2, and 3: **Low** | - Outlined dietary requirements for all patients pre [18F]FDG-PET/CT in methods section - States that 66.7% of patients underwent low-carb fat-allowed diet (does not specify which patients). |
|  | Outcomes 4 and 5: **Low** |  |
| Bias due to missing data. | Outcomes 1,2, and 3: **Moderate** | - Five participants were lost to follow up (data not included). - Data from all other patients were included in the primary analysis. - ‘Three months’ was measured from when antibiotics were stopped. |
|  | Outcomes 4 and 5: **Moderate** |  |
| Bias in the measurement of the outcome. | Outcomes 1, 2, and 3: **Low** | - Clear assessment through medical records. |
|  | Outcomes 4 and 5: **Serious** | - Clear outline of impacts of [18F]FDG-PET/CT (antibiotic changes), with two different interpreters assessing the medical records for this. - Clear outline of a true negative (with a timeline for this), and a false negative. - Did not provide information of treatment modifications in the ‘no [18F]FDG-PET/CT ’ control group. |
| Bias in selection of reported result. | Outcomes 1,2, and 3: **Moderate** | - Outcome of cause of death is mentioned in the methods, but not discussed in the results/discussion. - Outcome of relapse rate of SAB was clearly outlined in results. |
|  | Outcomes 4 and 5: **Moderate** |  |
| Overall risk of bias judgement | Outcomes 1,2, and 3: **Moderate**  Outcomes 4 and 5: **Serious** | |

**Article**: Berrevoets, 2019

Outcomes assessed:

1. Mortality at three months (specific to SAB).
2. Recurrent of infection at three months.

Assessors: AP, NS

| Risk of Bias Domain | Assessment | Comments |
| --- | --- | --- |
| Bias due to confounding. | **Serious** | - Appropriate use of Student’s t test and Χ^2^ test to compare variables, but no adjustment was made for these variables. - The study was conducted between two sites, with no information on how this was managed. - Same follow up period between groups, with no significant difference in duration of antibiotic treatment. |
| Bias in selection of participants. | **Moderate** | - Retrospective study with clear inclusion and exclusion criteria. - Immortal time bias was unlikely as the study included all with a positive SAB, however no adjustment was made. |
| Bias in classification of intervention. | **Moderate** | - Overall duration between recruitment and intervention was outlined, but not given for each specific patient. - Risk of outcome could have changed duration of antibiotic therapy. |
| Bias due to deviations from intended interventions. | **Low** | - Clear outline of dietary plan prior to [18F]FDG-PET/CT . - Method of reporting [18F]FDG-PET/CT results outlined, but does not state who by. |
| Bias due to missing data. | **Serious** | - Clear duration of follow up. - 12 patients were lost to follow up and therefore excluded, which may have biased results. |
| Bias in the measurement of the outcome. | **Low** | - Two independent physicians reviewed hospital records and determined causes of death. - Clear outcomes outlined. |
| Bias in selection of reported result. | **Moderate** | - Outcomes that were discussed in the initial protocol were discussed and outlined in the results section. |
| Overall risk of bias judgement | **Serious** | |

**Article**: Vos, 2010

Outcomes assessed:

1. Relapse of infection
2. Overall attributable mortality at three months and six months
3. Duration of antibiotic treatment
4. Number of diagnostic procedures to confirm [18F]FDG-PET/CT results

Assessors: AP, TB

| Risk of Bias Domain | Assessment | Comments |
| --- | --- | --- |
| Bias due to confounding. | Serious | - Use of a retrospective control group. There is a theoretical risk of bias if there was a change in assessment guidelines, but the authors commented that none occurred in this time. - All participants were followed up from a pre-specified point (first positive blood culture). - Matching done on pre-specified criteria, with ‘perfect matching’ for 102/115 patients (89%). The remaining 13 were imperfectly matched. Matching criteria did not include demographics or comorbidities. - Some significant differences between groups for risk factors for complicating infectious foci. - Carried out adjustment for potential confounding variables. |
| Bias in selection of participants. | **Moderate** | - Single-centre study. - Clear outline of selection criteria for participants, - Selection was done before the intervention was applied (prospective). - Intervention did not occur at the same time as follow up started. |
| Bias in classification of intervention. | **Moderate** | - [18F]FDG-PET/CT was specified as being required within two weeks of positive blood culture. The median and mean duration to [18F]FDG-PET/CT were seven and 6.8 days respectively. - 2 patients died before [18F]FDG-PET/CT performed, 2 refused consent. These 4 were all included in assessment. |
| Bias due to deviations from intended interventions. | **Moderate** | - Outline of preparation prior to [18F]FDG-PET/CT , but little information on diet before [18F]FDG-PET/CT . - Four patients assigned to [18F]FDG-PET/CT did not receive one (two did not consent, two died). They were matched and included in the analysis. |
| Bias due to missing data. | **Moderate** | - Four patients had data missing at six months (two from each group – not stated how this was managed. They were included in the three month data. |
| Bias in the measurement of the outcome. | **Moderate** | - Non-blinded study, but outcomes were clearly defined - [18F]FDG-PET/CT images evaluated by physicians without knowledge of prior clinical evaluation and imaging results (but were aware of intervention). - Clear methodology of [18F]FDG-PET/CT and reporting. |
| Bias in selection of reported result. | **Low** | - Discussed all outcomes as specified in methods. |
| Overall risk of bias judgement | **Serious** | |

**Article**: Ghanem-Zoubi, 2021

Outcomes assessed:

1. Mortality at 30 days, 90 days, and six months.
2. Relapse of infection.

Assessors: NS and TB

| Risk of Bias Domain | Assessment | Comments |
| --- | --- | --- |
| Bias due to confounding. | **Moderate** | - Single centre study. - Use of a historical matched cohort. - Cohorts were well matched on several characteristics, including age and comorbidities. - Regression analyses were done post-hoc for residual confounding. |
| Bias in selection of participants. | **Moderate** | - How participants were selected was well defined. - Adjusted for immortal time bias. |
| Bias in classification of intervention. | **Low** | - Clearly defined intervention - Small range for time to [18F]FDG-PET/CT (8-13 days), with a median of 11 days |
| Bias due to deviations from intended interventions. | **Moderate** | - Protocol for [18F]FDG-PET/CT was defined, but did not report adherence to this. - Co-interventions were not balanced between groups but analyses was appropriate |
| Bias due to missing data. | **Low** | - All patients have outcomes reported. - No information on handling of missing data (although none noted). |
| Bias in the measurement of the outcome. | **Low** | - Clearly defined outcomes, which were defined pre-trial. - [18F]FDG-PET/CT assessors were not blinded to the clinical history of patients. |
| Bias in selection of reported result. | **Moderate** | - Subgroup analyses were done post-hoc - Other results reported as specified in the study protocol |
| Overall risk of bias judgement | **Moderate** | |

**Article**: Yildiz 2019

Outcomes assessed:

1. Mortality at 30 days, 90 days, one year
2. Overall mortality
3. Detection of foci of infection on [18F]FDG-PET/CT .

Assessors: NS and TB

| Risk of Bias Domain | Assessment | Comments |
| --- | --- | --- |
| Bias due to confounding. | Moderate | - Single centre study - Two cohorts with similar baseline characteristics - Cases and controls were selected from the time period. - Little information on co-interventions. - Use of univariate and multivariate analyses |
| Bias in selection of participants. | **Moderate** | - Retrospective study, not randomised. - Well described selection of participants, based on characteristics observed before the intervention. - [18F]FDG-PET/CT was been performed at attending physician’s discretion. - No mention of adjustment for immortal time bias, but probably not required. |
| Bias in classification of intervention. | **Serious** | - Intervention timeframe is not entirely clear – described as having been done within one week, but the starting point was not specified. - No description on preparation for [18F]FDG-PET/CT . |
| Bias due to deviations from intended interventions. | **No information** | - No information on the quality of [18F]FDG-PET/CT , or whether local [18F]FDG-PET/CT preparation was adhered to. |
| Bias due to missing data. | **No information** | - No discussion of participants who were lost to follow up. |
| Bias in the measurement of the outcome. | **Low** | - Objective measures chosen for the outcome. |
| Bias in selection of reported result. | **Serious** | - Age related mortality reported but not described in protocol. - Values for mortality at each time point were not reported (only the p-values were given). - Overall mortality values were specified |
| Overall risk of bias judgement | **Serious** | |
